# Case of Intestinal Calculus

**Published:** 1827-12

**Authors:** 


### MISCELLANEOUS CASES.

### *Case of Intestinal Calculus.* Communicated by Mr. Wickham.

Master W—, the son of a medical man, was attacked, in the morning of October 17th, 1826, with a severe pain in the right lumbar region, just above the crista of the ileum, for which he took four grains of calomel and a cathartic draught. The pill remained on his stomach, but the draught was soon rejected. In the course of the night, the pain became more acute, and was accompanied by continued vomiting. No evacuation could be procured till the third or fourth day; previous to which, the abdomen had become tense and painful on pressure. The pain in the original seat of attack remained unabated. I need not say that bleeding, blisters, purgatives, and glysters were, in the course of the attack, suggested and employed by Dr. Littlehales, my father, and myself. The inflammatory symptoms pursued an uninterrupted course, and terminated the existence of the poor boy on the 23d.

By desire of the father and friends, the body was inspected, and the state of parts was as follows:—The general aspect of the abdomen was such as is usual after inflammation; the situation of the viscera natural. In the neighbourhood of the cœcum, there was effused a large quantity of adhesive matter and pus; the cœcum itself and the bowels lying by the side of it, very vascular. The appendix vermiformis cœci had an ulcerated opening in it, and a calculus was on the point of making its escape into the abdomen. This concretion was about the size of a cherry-stone; another, somewhat larger, was sacculated in the appendix, nearer to the body of the gut. The calculus has somewhat the appearance and consistence of wax, though harder, similar to that which is occasionally found to pass per anum; but whether biliary or intestinal, I cannot determine.

